# Both hand position and movement direction modulate visual attention

**DOI:** 10.3389/fpsyg.2013.00657

**Published:** 2013-10-01

**Authors:** Yariv Festman, Jos J. Adam, Jay Pratt, Martin H. Fischer

**Affiliations:** ^1^Division of Cognitive Sciences, University of PotsdamPotsdam, Germany; ^2^School of Mental Health and Neuroscience, University of MaastrichtMaastricht, Netherlands; ^3^Department of Psychology, University of TorontoToronto, ON, Canada

**Keywords:** embodied cognition, covert attention, hand dynamics, near-hand effect, perception

## Abstract

The current study explored effects of continuous hand motion on the allocation of visual attention. A concurrent paradigm was used to combine visually concealed continuous hand movements with an attentionally demanding letter discrimination task. The letter probe appeared contingent upon the moving right hand passing through one of six positions. Discrimination responses were then collected via a keyboard press with the static left hand. Both the right hand's position and its movement direction systematically contributed to participants' visual sensitivity. Discrimination performance increased substantially when the right hand was distant from, but moving toward the visual probe location (replicating the far-hand effect, Festman et al., 2013). However, this effect disappeared when the probe appeared close to the static left hand, supporting the view that static and dynamic features of both hands combine in modulating pragmatic maps of attention.

## Introduction

Our visual environment offers more information than we can process and act upon. Although the human visual system is characterized by extensive parallel processing, perception and action operate on one object at a time. Therefore, the ability to selectively attend to a portion of our visual environment is crucial for observers to compensate for their limited cognitive capacity. In the late 1980s, Rizzolatti et al. (1987) challenged the classical notion that selective attention is a structural control mechanism for selecting a certain portion of our visual space for prioritized processing. They proposed instead the “premotor theory of attention,” postulating that selective attention is driven by the same frontal-parietal circuits that are involved in the preparation of movements toward specific spatial locations. Accordingly, attentional selection was attributed to spatial pragmatic maps, which in turn depend on the preparation of goal-directed, spatially coded movements. Further studies of the coupling between eye movements and visual selection have found fairly supportive results for this idea (Hoffman and Subramaniam, 1995; Kowler et al., 1995; Deubel and Schneider, 1996; Fischer, 1999; Castet et al., 2006). For example, in a dual task paradigm that required a combination of target-directed saccade and letter-discrimination, performance was best when the discrimination target and saccade target referred to the same item (Deubel and Schneider, 1996).

While initial work explored the links between shifts of attention and oculomotor preparation, premotor theory asserts that the interplay between response programing and attentional selection is by no means restricted to oculomotor activity but can be driven by activity in pragmatic maps involved in programming hand movements (Rizzolatti et al., 1994). Unlike preparing an eye movement, which mainly involves goal selection, hand movement preparation also requires effector selection (i.e., left or right hand) and determining the dynamic position of the selected effector within different regions of space. Therefore, attention involved in preparing hand movements is likely affected by both hand selection and dynamic aspects of the response.

Studies of the association between attention and goal-directed hand movements have found inconsistent results. In a seminal study of the interplay between hand movements and visual selection, Tipper et al. (1992) studied the interference effect of a distractor stimulus while participants were engaged in goal-directed arm movements. They showed that the interference effect was present only when the distractor was located within the space between the start position of the hand and the location of the target, suggesting that visual attention extends from the start to the end position of the planned movement (see also Fischer, 1997). Other studies have instead shown that visual attention is strongly coupled to the response goals. Deubel et al. (1998) used a dual task paradigm to demonstrate that, when observers prepare a reaching movement to a certain location, performance is superior for targets displayed at the movement goal before movement onset. More recent work has revealed that, during the preparation of sequential reaching movements, attention is biased toward multiple goal-relevant locations in parallel (Baldauf et al., 2006). Similar parallel deployment of attention was also observed during the preparation of coordinated bimanual movements (Baldauf and Deubel, 2008). Taken together, these experimental studies suggest that motor preparation is coupled with visual selection of the intended goal locations *ahead* of the current position of the hand.

Complementing research on movement preparation, there is other work demonstrating that the static position of our hands can affect visual selection. Reed et al. (2006, 2010) studied whether the location of one's resting hand affects attentional selection. Participants placed one hand on a computer monitor and were faster in detecting probes near their hand (see also Adam et al., 2012; Gozli et al., 2012). Cosman and Vecera (2010) have shown that, in addition to prioritizing attention, the position of the observers' hand facilitates figure-ground segregation. Another demonstration of the influence of hand position on visual sensitivity has been revealed with a patient with a severe left hemianopsia. This patient exhibited improved detection of targets in his left visual field when his left arm was extended and placed near the target stimuli (Schendel and Robertson, 2004). Further support for distinct contributions of both the effector and the movement goal on visual selection has been obtained in evoked-response studies (Forster and Eimer, 2007; Gherri et al., 2007). Forster and Eimer (2007) cued participants to prepare movements of one hand (the effector) directed to touch the index finger of the opposite hand (the goal). Tactile probes were presented to the effector or the goal hand during movement preparation. Somatosensory ERPs to these tactile probes were larger when probes were presented to the effector than when presented to the goal hand, suggesting that attentional engagement was stronger for the effector than for the movement goal. Together, these studies suggest that proprioceptive signals guide attention toward locations *near* the hand which may facilitate the interaction with objects. This notion gains support from physiological recording in non-human primates which found visuo-tactile neurons that respond to the combined visual and somatosensory feedback from the body (Graziano and Gross, 1998; see also Andersen et al., 1997, for a review).

The studies reviewed so far have generally segmented the normally continuous stream of movement into discrete units of analysis. In other words, the focus has been on static hand postures or single actions. This convenience-driven methodological practice limits our knowledge about attention deployment during continuous movements in more realistic tasks, such as manipulating hand-held devices. Recently, a few studies have re-examined the online influence of action on perception (for recent reviews, see Tseng et al., 2012; Brockmole et al., 2013). One such example is Adam et al. (2012), who studied the effect of hand proximity on letter identification performance while participants adopted a bimanual posture (static) or performed a movement (dynamic) underneath a display. Results confirmed and extended earlier findings of improved probe identification near the hand (the “near-hand effect”) to bimanual continuous movements. In contrast to this result, using a single hand movement, letter discrimination was best when the hand was far from and moved toward the probe (a “far-hand effect”; Festman et al., 2013). Both studies converge on the view that proprioceptive information regarding the current hand position can affect the distribution of spatial attention during the execution of hand movements. However, it remains unclear how the near-hand effect and the far-hand effect together influence visual selection during continuously changing hand movements.

To examine the interplay of near- and far-hand effects, we combined visually concealed continuous hand movements (Adam et al., 2012; Festman et al., 2013) with an attentionally demanding letter discrimination task (Braun and Julesz, 1998) that was presented contingent upon the course of hand motion. Our participants were required to move their (concealed) right hand back and forth, from side to side, under a display, while their static (visible) left hand was next to a keyboard to the left of the display. During the hand movement, a brief visual probe stimulus appeared contingent upon the right hand passing through one of six positions. In this experimental design both the near- and the far-hand effect are likely to modulate visual selection. We hypothesized that if the near-hand effect (hand proximity) is dominated by static hand posture, it will facilitate selection to the left side of the display (i.e., improve performance for attentional probes presented left of fixation). In contrast to that, if the far-hand is driven by dynamic hand motion, as revealed in our recent study (Festman et al., 2013), it will facilitate or attenuate selection depending on movement direction. If selection in the left side of the display is facilitated by the nearby presence of the left hand, the far-hand effect should have an impact mainly on the right side of the display (i.e., modulating performance for attentional probes presented to the right of fixation).

## Methods

### Participants

A convenience sample of five participants (age: 20–27, 2 male, all right handed) with normal or corrected-to-normal vision participated in the experiment. They gave written informed consent and were paid for their participation.

### Apparatus

**Figure 1:**
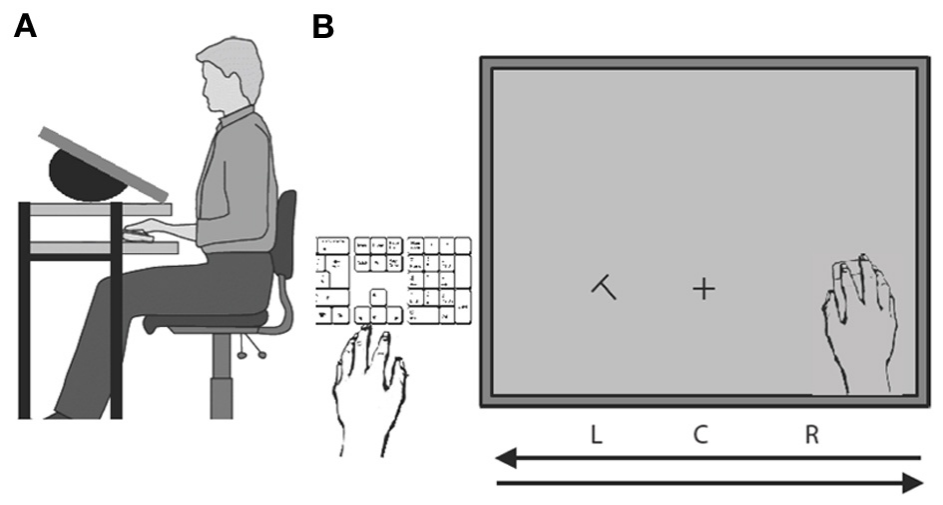
**Schematic illustration of the experimental setup**. **(A)** Side view. **(B)** Bird's eye view of the two hands and their respective tasks. The right hand was always moving from right to left and back on a shelf under the display. Participants discriminated a probe letter (T or L) that was briefly displayed to the left or right of a fixation cross and was followed by an F-shape mask (not illustrated). Probes were displayed when the right hand reached positions R, C, or L during either leftward or rightwards movement. After motion completion the participant indicated the probe's identity by keyboard press with the visible left hand.

Participants were seated in front of a two-layered computer-desk. Their left hand was placed near the lower right side of a keyboard placed left of display. Their right hand was placed on the shelf below a 22 inch LCD screen (65 × 41° usable field of view), which was set on the top layer of the desk, with an angle of 30° to the horizon (see Figure 1A). When viewing the screen from above (viewing distance 35 cm), the right hand was invisible to the participants. Hand position was monitored via a single-button Apple optical computer mouse that was held by the right hand and allowed hand-position contingent probe onsets. Mouse speed was matched to that of hand speed, so that the cursor position (hidden from observers) was always contingent with hand position. Mouse acceleration was disabled. The keyboard was used for recording participants' responses with their left hand.

### Stimuli

The experiment was programed and controlled in Matlab. All stimuli were generated by using the Psychophysical Toolbox (Brainard, 1997; Pelli, 1997). The attentional probe was a rotated T or L (size: 2.4 × 2.4°, eccentricity: 10.3°) that was presented either to the left (L) or to the right (R) of a fixation cross (size: 2 × 2°) that was shown continuously 6° below the display center (position C). After an individually adjusted stimulus onset asynchrony (SOA) the probe was followed by an F-shaped mask at the same location (Figure 1B).

### Procedure

On every trial, participants were required to move their hand once from the right side to the left side under the computer screen and back (thus covering a distance of 45 cm twice). Before each movement, two short audio tones (1200 Hz) were played with an interval of 1200 ms, used for both cuing participants to initiate the hand movement and indicating the time from the start to the reverse of the movement, thus prescribing a movement speed of 37.5 cm/s.

During the hand movement, the visual probe was presented briefly, followed by a mask. In order to prevent a direct fixation on the probe, we used short SOAs (typically <150 ms) that were individually adjusted through an adaptive staircase procedure. On each trial, the probe was displayed either in the lower left or in the lower right location of the screen with one of six equiprobable onset times: The probe appeared either with the hand reaching position R, C, or L while moving to the left side of the screen or with the hand reaching position L, C, or R while moving back toward the starting position under the right edge of the screen (Figure 1B). After movement completion, participants indicated the probe's identity via a keyboard press with their left hand. Pretests established that onset delays were minimized to one frame and this was the same in all conditions. If a larger delay occurred, this was registered and the trial was discarded.

There were a total of 24 different trial conditions (2 probe positions × 2 letter probes × 6 hand positions). Each block consisted of 30 trials: 24 trials with probe presentation (1trial per condition) and 6 additional trials without probes. This paradigm enables the examination of the influence of both hand proximity (near-hand effect) and hand movement direction (movement direction effect) on the allocation of covert attention.

Participants were trained for at least 1–2 h on performing the hand motion and probe discrimination task before data collection. Participants started with an SOA value of 250 ms that was either decreased or increased by 50 ms if performance in the previous block exceeded 85% correct discriminations or undercut 65% correct discriminations, respectively. The training ended when participants performed probe identification at 75% correct with SOA values <200 ms. However, since participants' performance could further improve, this staircase procedure continued during testing. Each participant was tested for 3–5 h each, on separate days over a period of 1–2 weeks. This resulted in 900–1200 trials per participant.

## Results

**Figure 2:**
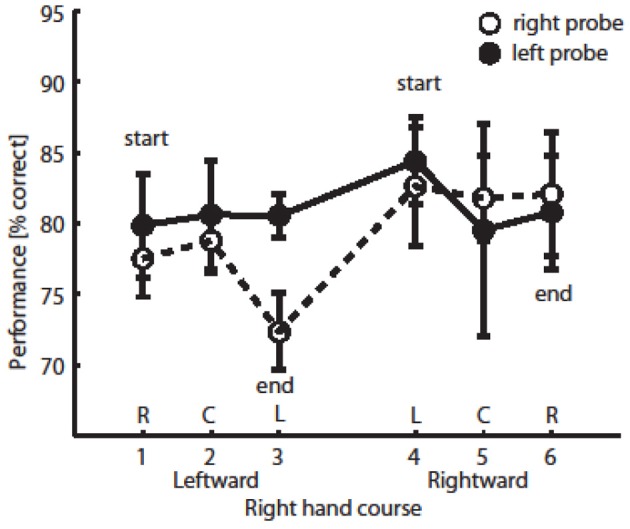
**Probe discrimination performance**. Performance on trials with right probe location (open circles) or left probe location (full circles), depending on hand position (x-axis, proportional to time on trial). Each circle denotes average performance (with SE).

Data were filtered as follows; experimental trials with movement times <1.4 or >3.0 s or with SOAs >220 ms were excluded to ensure homogeneity of performance and to prevent contamination from probe-directed eye movements (2% of all the data). Average movement time was 2.311 s (*SD* = 0.083), and average SOA was 137 ms (*SD* = 18). Mean probe discrimination performance across participants as a function of the time course of hand position (along the x-axis) is shown in Figure 2, separately for the two probe locations.

The data were analyzed separately for left and right probe locations because this factor did not interact with any other factor in a 3-Way ANOVA (this was also the case in the previous published study with a larger sample size, see Festman et al., 2013). Given our prediction of a selective effect of hand placement, separate repeated measure analyses of variance (ANOVA) were conducted for the two probe locations (left and right side of the display) on the mean performance in probe discrimination, with hand position (six levels) as within-subjects variable. We found that when the probe was presented to the right of fixation, there was a significant effect on hand position on discrimination performance [*F*_(5, 20)_ = 3.704, *p* < 0.05; see Figure 2 open circles] (*M* = 77.5, 78.8, and 72.3% for the R, C, and L positions, correspondingly, when participants moved their hand leftward and 82.6, 81.8, and 82.1% for L, C, and R positions, correspondingly, when they moved their hand rightward during the latter part of the motion course). However, when the probe was presented to the left of fixation, there was no effect of hand position during the movement on discrimination performance [*F*_(5, 20)_ = 0.339, *p* > 0.75; see Figure 2 full circles].

**Figure 3:**
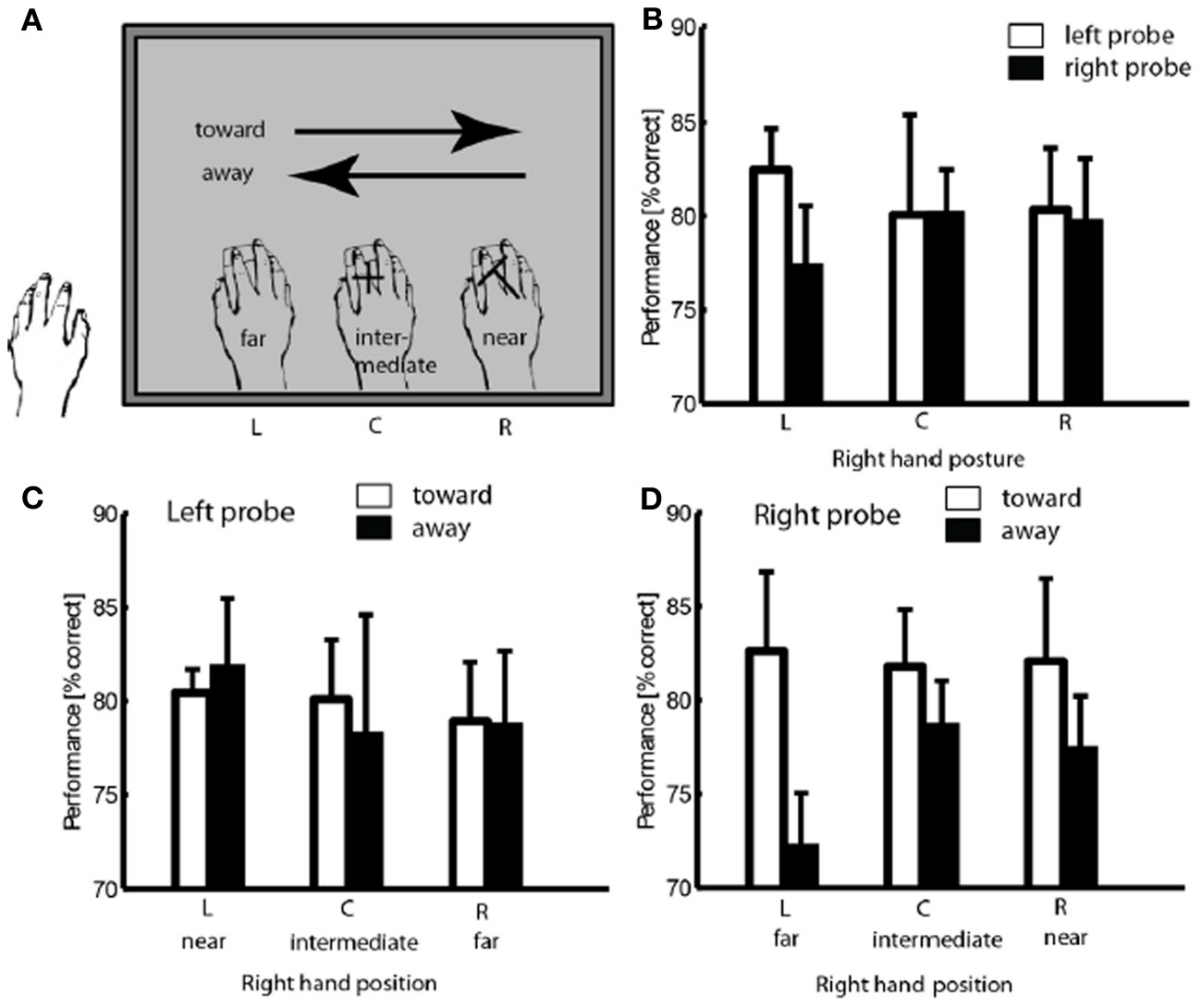
**Illustration of trial classification, using a trial with probe at position R as example (A)**. Performance in different hand positions for two probe locations. **(B)** Performance in discrimination left and right probe as a function of hand position and movement direction **(C,D)** Error bars show standard error of the mean.

The main effect of right hand position did not reach significance [*F*_(2, 8)_ = 0.685]. We found a trend for the interaction of hand position and probe location; When participants' hand was under the left side of the display (position L), mean performance was higher in trials with left probe compared to trials with right probe [82.5 vs. 77.5%, *F*_(2, 8)_ = 4.056, *p* = 0.11; see Figure 3B].

The participants were instructed to perform two continuous smooth hand movements from side to side in every trial. The turning point under the left edge of the screen was therefore an endpoint of leftward movement and a start point of rightward movement, just as the point under the right edge of the screen served as a start point for leftward movements and end point for rightward movements. A 2-factor repeated measures analysis of variance (ANOVA) was conducted to evaluate effects of movement latency (start, intermediate, end) and movement direction (leftward, rightward) on perceptual performance. Neither the main effects nor the interaction reached significance [*F*_(2, 8)_ < 1], suggesting that the turning point does not induce an effect on attentional allocation.

Trials were then classified with regard to the proximity between probe location and hand proximity (near, intermediate, and far) and with regard to the direction of hand movement relative to the probe position (i.e., leftward hand movements were defined as movement *toward* the left probe and *away* from the right probe, and vice versa for rightward hand movements; see Figure 3A). Separate 2-factors repeated measure analyses of variance (ANOVA) were conducted for the two probe locations on the mean performance in probe discrimination, with hand proximity (three levels) and movement direction (two levels) as within-subjects variables. We found a significant interaction of hand proximity and movement direction when the probe was presented to the right of fixation; mean performance was significantly higher when the participants' hand was under the left side of the display (far proximity) moving toward it (rightwards), compared to when moving away from it (leftwards) [82.6 vs. 72.3%, *F*_(2, 8)_ = 4.494, *p* < 0.05, see Figure 3D]. No significant effect of movement direction was found in trials with left probe [*F*_(1, 4)_ = 0.348, see Figure 3C].

## Discussion

The present study examined the combined effect of a static (left) hand and dynamic (right) hand on visual discrimination performance. Our findings revealed a strong modulation in performance by the direction of hand movement which is strongest when the moving hand is far from the attentional probe and moving toward it (replicating the “far-hand effect”; Festman et al., 2013). However, probes presented to the left of fixation were not affected by this far-hand effect, suggesting that the nearby (static) presence of the left hand eliminated the far-hand effect for left side probes.

The present result helps to clarify the apparent difference between the “near-hand effect” of Adam et al. (2012) and the “far-hand effect” of Festman et al. (2013). Specifically, the bimanual counterpace movement task of Adam et al. brought one hand in the vicinity of the other hand as the two hands moved together, thus shrinking the size of the attentional pragmatic map. In contrast, participants in Festman et al.'s earlier study used only a single hand, thus allowing for a shift of the entire pragmatic map toward far probe locations, which enhanced visual selectivity there. The present study, by simply placing observers' other hand near the turning point of the hand movement, reduced the resulting far-hand effect again, therefore suggesting that attentional pragmatic maps are dynamically delimited by the current positioning of both hands, as was proposed by Rizzolatti et al. (1994).

Recently it has been suggested that visual processing is altered near the hands. Gozli et al. (2012) found that placing the hands near the display improves performance in temporal tasks, while attenuating performance in spatial tasks. In the current study, the letter discrimination task demands both spatial and temporal detection. Hand movements toward or away from the probe letter appear to facilitate or attenuate visual processing, respectively. However, the nature of the task we employed does not reveal whether magno- or parvo-cellular processing is affected.

Our initial result does not clarify whether the modulating effect of the left hand is driven by its visual or proprioceptive cues. While future work should investigate this point, we refer readers to the work of Reed et al. (2006, Experiments 2 and 3) which suggests that hand proprioception is sufficient to modulate visuo-spatial attention near the hand.

Our findings are consistent with a bimodal neuronal integration mechanism that processes both visual information and motor feedback (efference copy signals) from the body (Graziano and Gross, 1998). This, in turn, provides an online, multisensory representation of visual information in peri-personal space centered on active body parts (see Graziano and Gross, 1998; Graziano, 2001) and is also involved in directing spatial attention (Bremmer et al., 2001; Halligan et al., 2003). This bimodal integration mechanism has been made responsible for earlier findings of a near-hand advantage for visual attention in search, detection, and attentional blink tasks (cf. Abrams et al., 2008). More recently, it has also been proposed to account for the modulating effects of hand position in flanker interference tasks (Davoli and Brockmole, 2012).

To summarize, our movement-contingent attentional probing method is capable of discovering the combined impact of both static and dynamic hand positions on visual attention deployment. Further studies of this proposed mechanism may expand our understanding of information uptake in real-life situations, such as swiping movements and other manual interactions with hand-held devices—for example, smart phones and tablet PCs (Dufau et al., 2011; Miller, 2012).

### Conflict of interest statement

The authors declare that the research was conducted in the absence of any commercial or financial relationships that could be construed as a potential conflict of interest.

## References

[B1] Abrams R. A. Davoli C. C. Du F. Knapp W. H. 3rd. Paull D. (2008). Altered vision near the hands. Cognition 107, 1035–1047 10.1016/j.cognition.2007.09.006 17977524

[B2] Adam J. Bovend'Eert T. van Dooren F. Fischer M. Pratt J. (2012). The closer the better: hand proximity dynamically affects letter recognition accuracy. Atten. Percept. Psychophys. 74, 1533–1538 10.3758/s13414-012-0339-3 22777734 PMC3447143

[B3] Andersen R. A. Snyder L. H. Bradley D. C. Xing J. (1997). Multimodal representation of space in the posterior parietal cortex and its use in planning movements. Annu. Rev. Neurosci. 20, 303–330 10.1146/annurev.neuro.20.1.303 9056716

[B4] Baldauf D. Deubel H. (2008). Visual attention during the preparation of bimanual movements. Vision Res. 48, 549–563 10.1016/j.visres.2007.11.023 18206205

[B5] Baldauf D. Wolf M. Deubel H. (2006). Deployment of visual attention before sequences of goal-directed hand movements. Vision Res. 46, 4355–4374 10.1016/j.visres.2006.08.021 17034829

[B6] Brainard D. H. (1997). The psychophysics toolbox. Spat. Vis. 10, 433–436 10.1163/156856897X00357 9176952

[B7] Braun J. Julesz B. (1998). Withdrawing attention at little or no cost: detection and discrimination tasks. Percept. Psychophys. 60, 1–23 10.3758/BF03211915 9503909

[B8] Bremmer F. Schlack A. Duhamel J. R. Graf W. Fink G. R. (2001). Space coding in primate posterior parietal cortex. Neuroimage 14, S46–S51 10.1006/nimg.2001.0817 11373132

[B9] Brockmole J. R. Davoli C. C. Abrams R. A. Witt J. K. (2013). The world within reach: the effect of hand posture and tool use on visual cognition. Curr. Dir. Psychol. Sci. 22, 38–44 10.1177/0963721412465065

[B10] Castet E. Jeanjean S. Montagnini A. Laugier D. Masson G. S. (2006). Dynamics of attentional deployment during saccadic programming. J. Vis. 6, 196–212 10.1167/6.3.2 16643090

[B11] Cosman J. D. Vecera S. P. (2010). Attention affects visual perceptual processing near the hand. Psychol. Sci. 21, 1254–1258 10.1177/0956797610380697 20713634

[B12] Davoli C. C. Brockmole J. R. (2012). The hands shield attention from visual interference. Atten. Percept. Psychophys. 74, 1386–1390 10.3758/s13414-012-0351-7 22855428

[B13] Deubel H. Schneider W. X. (1996). Saccade target selection and object recognition-evidence for a common attentional mechanism. Vision Res. 36, 1827–1837 10.1016/0042-6989(95)00294-4 8759451

[B14] Deubel H. Schneider W. X. Paprotta I. (1998). Selective dorsal and ventral processing: evidence for a common attentional mechanism in reaching and perception. Vis. Cogn. 5, 81–107 10.1080/713756776

[B15] Dufau S. Dunabeitia J. A. Moret-Tatay C. McGonigal A. Peeters D. Xavier-Alario F. (2011). Smart phone, smart science: how the use of Smartphones can revolutionize research in cognitive science. PLoS ONE 6:e24974 10.1371/journal.pone.0024974 21980370 PMC3182196

[B16] Festman Y. Adam J. Pratt J. Fischer M. (2013). Continuous hand movement induces a far-hand bias in attentional priority. Atten. Percept. Psychophys. 75, 644–649 10.3758/s13414-013-0430-4 23404523

[B17] Fischer M. H. (1997). Attention allocation during manual movement preparation and execution. Eur. J. Cogn. Psychol. 9, 17–51 10.1080/713752546

[B18] Fischer M. H. (1999). An investigation of attention allocation during sequential eye movement tasks. Q. J. Exp. Psychol. 52A, 649–677 10.1080/713755838 10504902

[B19] Forster B. Eimer M. (2007). Covert unimanual response preparation triggers attention shifts to effectors rather than goal locations. Neurosci. Lett. 419, 142–146 10.1016/j.neulet.2007.04.027 17485166

[B20] Gherri E. Van Velzen J. Eimer M. (2007). Dissociating effector and movement direction selection during the preparation of manual reaching movements: evidence from lateralized ERP components. Clin. Neurophysiol. 118, 2031–2049 10.1016/j.clinph.2007.06.003 17646131 PMC2386665

[B21] Gozli D. G. West G. L. Pratt J. (2012). Hand position alters vision by biasing processing through different visual pathways. Cognition 124, 244–250 10.1016/j.cognition.2012.04.008 22633129

[B22] Graziano M. S. A. (2001). A system of multimodal areas in the primate brain. Neuron 29, 4–6 10.1016/S0896-6273(01)00174-X 11182075

[B23] Graziano M. S. A. Gross C. G. (1998). Spatial maps for the control of movement. Curr. Opin. Neurobiol. 8, 195–201 10.1016/S0959-4388(98)80140-2 9635202

[B24] Halligan P. W. Fink G. R. Marshall J. C. Vallar G. (2003). Spatial cognition: evidence from visual neglect. Trends Cogn. Sci. 7, 125–133 10.1016/S1364-6613(03)00032-9 12639694

[B25] Hoffman J. E. Subramaniam B. (1995). The role of visual attention in saccadic eye movements. Percept. Psychophys. 57, 787–795 10.3758/BF03206794 7651803

[B26] Kowler E. Anderson E. Dosher B. Blaser E. (1995). The role of attention in the programming of saccades. Vision Res. 35, 1897–1916 10.1016/0042-6989(94)00279-U 7660596

[B27] Miller G. F. (2012). The smartphone psychology manifesto. Perspect. Psychol. Sci. 7, 221–237 10.1177/1745691612441215 26168460

[B28] Pelli D. G. (1997). The videotoolbox software for visual psychophysics: transforming numbers into movies. Spat. Vis. 10, 437–442 10.1163/156856897X00366 9176953

[B29] Reed C. L. Betz R. Garza J. Roberts R. (2010). Grab it! Biased attention for functional hand and tool space. Atten. Percept. Psychophys. 72, 236–245 10.3758/APP.72.1.236 20045892

[B30] Reed C. L. Grubb J. D. Steele C. (2006). Hands up: attentional prioritization of space near the hand. J. Exp. Psychol. Hum. Percept. Perform. 32, 166–177 10.1037/0096-1523.32.1.166 16478334

[B31] Rizzolatti G. Riggio L. Dascola I. Umiltà C. (1987). Reorienting attention across the horizontal and vertical meridians: evidence in favor of a premotor theory of attention. Neuropsychologia 25, 31–40 10.1016/0028-3932(87)90041-8 3574648

[B32] Rizzolatti G. Riggio L. Sheliga B. M. (1994). Space and selective attention, in Attention and Performance XV, eds Umiltà C. Moscovitch M. (Cambridge, MA: MIT Press), 231–265

[B33] Schendel K. Robertson L. C. (2004). Reaching out to see: arm position can attenuate human visual loss. J. Cogn. Neurosci. 16, 935–943 10.1162/0898929041502698 15298781

[B34] Tipper S. P. Lortie C. Baylis G. C. (1992). Selective reaching: evidence for action centered attention. J. Exp. Psychol. Hum. Percept. Perform. 18, 891–905 10.1037/0096-1523.18.4.891 1431753

[B35] Tseng P. Bridgeman B. Juan C. H. (2012). Take the matter into your own hands: a brief review of the effect of nearby-hands on visual processing. Vision Res. 72, 74–77 10.1016/j.visres.2012.09.005 23010259

